# Rapid colistin susceptibility determination by a novel fluorogenic method in *Klebsiella pneumoniae* strains

**DOI:** 10.3389/fmicb.2025.1693119

**Published:** 2026-01-09

**Authors:** Meliha Negin, Meltem Ayas, Isin Akyar, Erkan Mozioglu, Tanil Kocagoz

**Affiliations:** 1 Department of Medical Biotechnology, Institute of Health Sciences, Acibadem Mehmet Ali Aydinlar University, Istanbul, Türkiye; 2 School of Pharmacy, Department of Pharmaceutical Technology, Istanbul Medipol University, Istanbul, Türkiye; 3 Department of Medical Laboratory Techniques, Vocational School of Health Services, Acibadem Mehmet Ali Aydinlar University, Istanbul, Türkiye; 4 Department of Medical Microbiology, School of Medicine, Acıbadem Mehmet Ali Aydınlar University, Istanbul, Türkiye

**Keywords:** colistin resistance, fluorometric assay, *Klebsiella pneumoniae*, minimum inhibitory concentration, rapid antimicrobial susceptibility testing

## Abstract

**Introduction:**

*Klebsiella pneumoniae* is a causative agent that can lead to fatal infections, especially in intensive care patients. Colistin treatment is employed as a last resort in *K. pneumoniae* infections caused by strains resistant to almost all antibiotics from different groups. However, in recent years, colistin resistance has also become widespread in *K. pneumoniae* strains. Therefore, before starting treatment with this relatively toxic drug, it is essential to know its susceptibility to colistin. A standard colistin susceptibility test typically takes 24–48 h. In this study, we investigated the accuracy of our novel fluorometric method, which enables the determination of colistin minimal inhibitory concentration (MIC) within 2 h for *K. pneumoniae*.

**Methods:**

For this purpose, we determined and compared the MIC values of 24 colistin-resistant and 18 colistin-susceptible *K. pneumoniae* strains using the standard broth microdilution method and the new fluorometric method.

**Results:**

In the comparison of MIC values determined by standard and fluorometric methods, it was observed that the MIC values of 22 strains were identical, there was a 1-dilution (1/2) difference in 14 strains, and a 2-dilution (1/4) difference in six strains.

**Discussion:**

We believe that the new fluorometric antibiotic susceptibility testing method will be a valuable tool in the rapid detection of colistin resistance in *K. pneumoniae* and thus for guiding appropriate treatment in critically ill patients.

## 1 Introduction

Carbapenemase-producing *K. pneumoniae* (CPKP) has become a globally recognized cause of nosocomial infections, including pneumonia, bloodstream infections, wound or surgical site infections, and meningitis. The emergence and dissemination of carbapenem resistance among *K. pneumoniae* strains represent a serious worldwide threat and requires urgent public health attention. These organisms can cause infections with high mortality rates and have the potential to spread widely (Centers for Disease Control and Prevention (CDC), 2015). In addition, increasing resistance to all the last-resort antibiotics, including colistin, is a serious cause for concern (Giordano and Barnini, 2018).

Carbapenem resistance (CR) refers to the ability of bacteria to survive and grow in the presence of clinically relevant concentrations of carbapenems. The US CDC defines carbapenem-resistant Enterobacteriaceae (CRE) as bacteria non-susceptible to any carbapenem (i.e., showing an MIC of 4 mg/L or higher for doripenem, meropenem, or imipenem or 2 mg/L or higher for ertapenem) or documented to produce a carbapenemase (Durante-Mangoni et al., 2019).

Antimicrobial resistance has emerged as one of the most pressing global health challenges, particularly in relation to multidrug-resistant Gram-negative bacteria, which are responsible for severe hospital-acquired infections and belong to the ESKAPEE (*Enterococcus faecium, Staphylococcus aureus, K. pneumoniae, Acinetobacter baumannii, Pseudomonas aeruginosa, Enterobacter* spp., and *Escherichia coli*) group (Karampatakis et al., 2025). Colistin has often been used as a therapeutic option for the treatment of carbapenem-resistant *K. pneumoniae* infections. However, the imprudent use of colistin has caused the rapid spread of colistin resistance in *K. pneumoniae* producing carbapenemase enzymes, particularly the KPC-type carbapenemases (Giordano and Barnini, 2018; Hindler and Humphries, 2013). Additionally, colistin use in agriculture and animal husbandry has contributed to the spread of colistin resistance (Stefaniuk and Tyski, 2019).

Since *K. pneumoniae* causes life-threatening infections in critically ill patients, early determination of colistin susceptibility and appropriate treatment may be life-saving. This situation indicates the need for the development of accurate, rapid, and reliable methods for detecting colistin resistance (Lee et al., 2016).

Recently, it has become clear that susceptibility testing for colistin is a major challenge (Monaco et al., 2014), and semi-automated systems, commonly used in microbiology laboratories for antimicrobial susceptibility testing, result in a high proportion of very major discrepancies and are unsuitable for detecting colistin-resistance (Park et al., 2016; Reller et al., 2009). Hence, the broth microdilution (BMD) assay has been the only recommended antimicrobial susceptibility testing method by international committees for *K. pneumoniae*, concluding that BMD represents the reference method for colistin antimicrobial susceptibility testing. Several factors that affect colistin-susceptibility results have been evaluated, but no alternative method to BMD has been proposed so far (Stefaniuk and Tyski, 2019). The availability of a rapid, reliable, and inexpensive method that allows the screening of colistin-resistant isolates in routine diagnostic laboratories with a high proportion of MDR Gram-negative bacteria is consequently an urgent clinical need (Giordano and Barnini, 2018).

In this study, we have developed and evaluated a new rapid antibiotic susceptibility testing method, based on the increase of fluorescence of a fluorescein depending on oxygen radicals produced by bacteria. Reactive oxygen species (ROS) are oxygen derivatives and damage biomolecules such as proteins and DNA. To overcome the negative effects of superoxide toxicity, most bacteria surviving in aerobic environments encode an enzyme named superoxide dismutase (SOD), converting the superoxide radical into the less toxic H_2_O_2_ and water. In the *K. pneumoniae* genome, there are three superoxide dismutase genes, sodA, sodB, and sodC, encoding SODs having Mn-, Fe-, and CuZn-cofactors, respectively (Najmuldeen et al., 2019). When excited by light in visible light wavelength, some fluorescent chemicals produce superoxide radicals; it acts as a kind of oxidase enzyme. Hydrogen peroxide produced by SOD activity oxidizes these molecules, resulting in higher fluorescence (Liu et al., 2018). In this study, we took advantage of this photocatalytic property of fluorescein, a phenomenon that has been used as a biosensor to determine the activity of many enzymes. We showed that this phenomenon, related to the higher fluorescence of molecules oxidized with hydrogen peroxide, can be used to detect bacterial vitality in the presence of antibiotics. Hydrogen peroxide produced by the SOD enzymes of living bacteria plays a key role in this purpose (Odyniec et al., 2020).

By using this property, we have developed a novel fluorogenic method that can determine the antibiotic susceptibility of Enterobacteriaceae within 2 h (Bio-T Biotechnology Solutions, Istanbul, Turkiye. Patent pending). We have evaluated the reliability of this method in the determination of colistin MIC in *K. pneumoniae* strains, which may be one of the most important applications of this new technique.

## 2 Materials and methods

### 2.1 Bacterial isolates

A total of 42 *K. pneumoniae* collection strains previously isolated from clinical samples were included in the study. Among these strains, 28 were previously identified as colistin-resistant and 14 as colistin-susceptible. The species of the strains was identified as *K. pneumoniae* using MALDI-TOF MS (Bruker Daltonik GmbH, Bremen).

### 2.2 Rapid MIC determination by the fluorometric method

Bacterial strains, stored at −20 °C in stock culture media, were grown overnight at 37 °C on Mueller-Hinton Agar (BioLife) plates. Bacteria from a single colony were suspended in Muller-Hinton Broth (Oxoid), adjusted to a final turbidity of 0.5 McFarland. This suspension was used directly for MIC determination by the fluorogenic method and diluted 1:100 times in Mueller-Hinton Broth for use in the reference method defined by the European Committee on Antimicrobial Susceptibility Testing (EUCAST) for colistin MIC determination (EUCAST (European Committee on Antimicrobial Susceptibility Testing), 2016).

Dilutions of colistin (Colistin Sulfate Salt, SIGMA, C4461-1G) were prepared in 96-well microtiter plates to obtain final concentrations ranging from 128 μg/mL to 0.25 μg/mL as the final concentration of colistin after the addition of inoculum. The last two wells on each row that did not contain colistin were used as growth control and medium control, respectively. We added 50 μL of a 0.5 McFarland bacterial suspension prepared in fluorometric medium, Mueller Hinton broth supplemented with fluorescein, to each well except the medium control well. Fifty microliters of the fluorometric medium without bacteria was added to the medium control well. The microplate was incubated at 37 °C for 3 h in the fluorometer (Varioskan Multimode Microplate Reader, Thermo Fisher Scientific), and fluorescence was continually monitored.

For the quality control of the Mueller Hinton medium and colistin antibiotic, colistin-susceptible *Escherichia coli* ATCC 25922 and colistin-resistant *Escherichia coli* NCTC 13846 were used. The results were interpreted using the EUCAST Clinical Breakpoint and reported as susceptible for ≤2 μg/mL and resistant for >2 μg/mL, as recommended for Enterobacterales strain in EUCAST (European Committee on Antimicrobial Susceptibility Testing) (2025).

### 2.3 Statistical analysis for diagnostic performance metrics

For the statistical analysis was performed using the following formulas:


Sensitivity:TP/(TP+FN)

Specificity:TN/(FP+TN)

Positive Predictive Value(PPV):TP/(TP+FP)

Negative Predictive Value(NPV):TN/(TN+FN)

The technical agreement was evaluated based on essential MIC agreement, defined as MIC values within ±1 dilution of the MIC mode (Gütgemann et al., 2023). MIC values were compared between the two methods regarding the susceptibility profile (S or R), and the technical agreement rate was calculated using the formula:


$$100^{*}\;(\begin{array}{l} \text{Number of Results}\;\mathrm{by}\;\text{Fluorometric Method}/\\ \text{Number of Results}\;\mathrm{by}\;\text{Standard Method} \end{array}).$$

## 3 Results

### 3.1 Colistin MIC values by the standard and fluorometric methods

**Figure 1:**
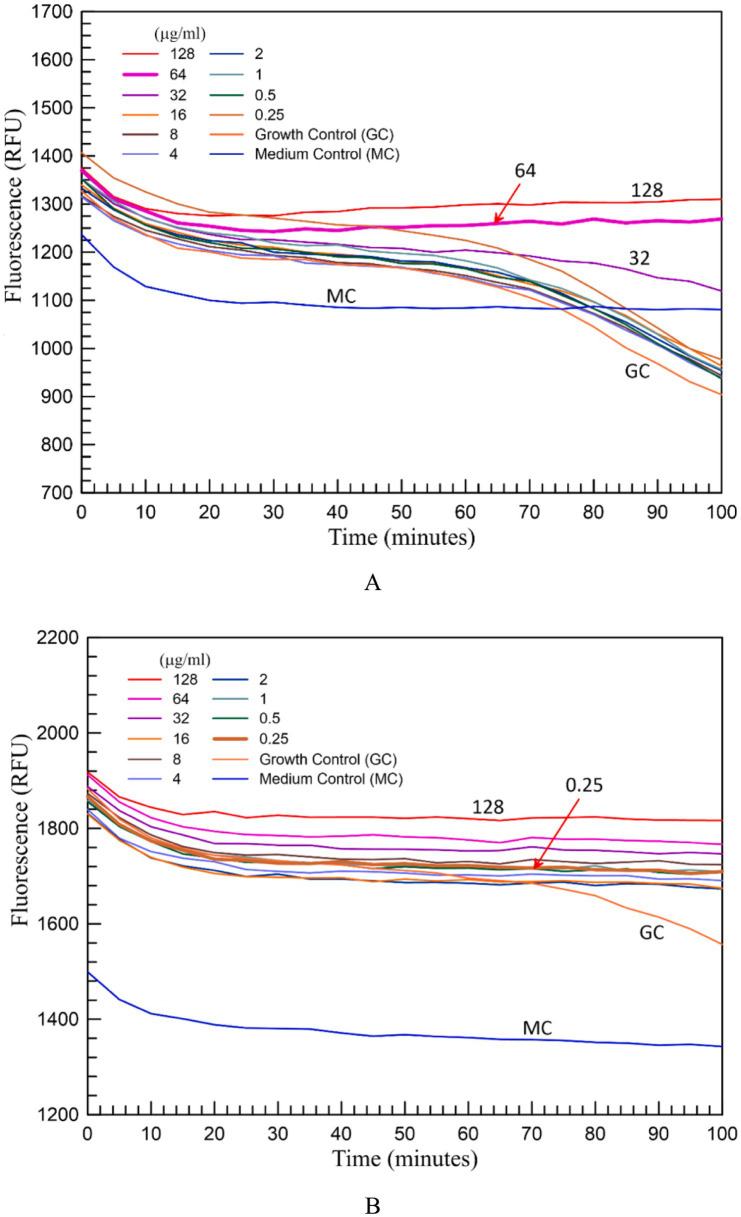
The growth curves of a colistin-resistant **(A)** and colistin-susceptible **(B)** *K. pneumoniae* strain before data transformation. The MIC values are indicated by an arrow. In colistin resistant strain **(A)** a drop in fluorescence is seen up to 32 μg/mL of colistin concentration indicating growth of bacteria in these wells. The MIC was determined as 64 μg/mL. In the susceptible strain there was only growth in growth control well indicating that MIC was below 0.25 μg/mL.

**Figure 2:**
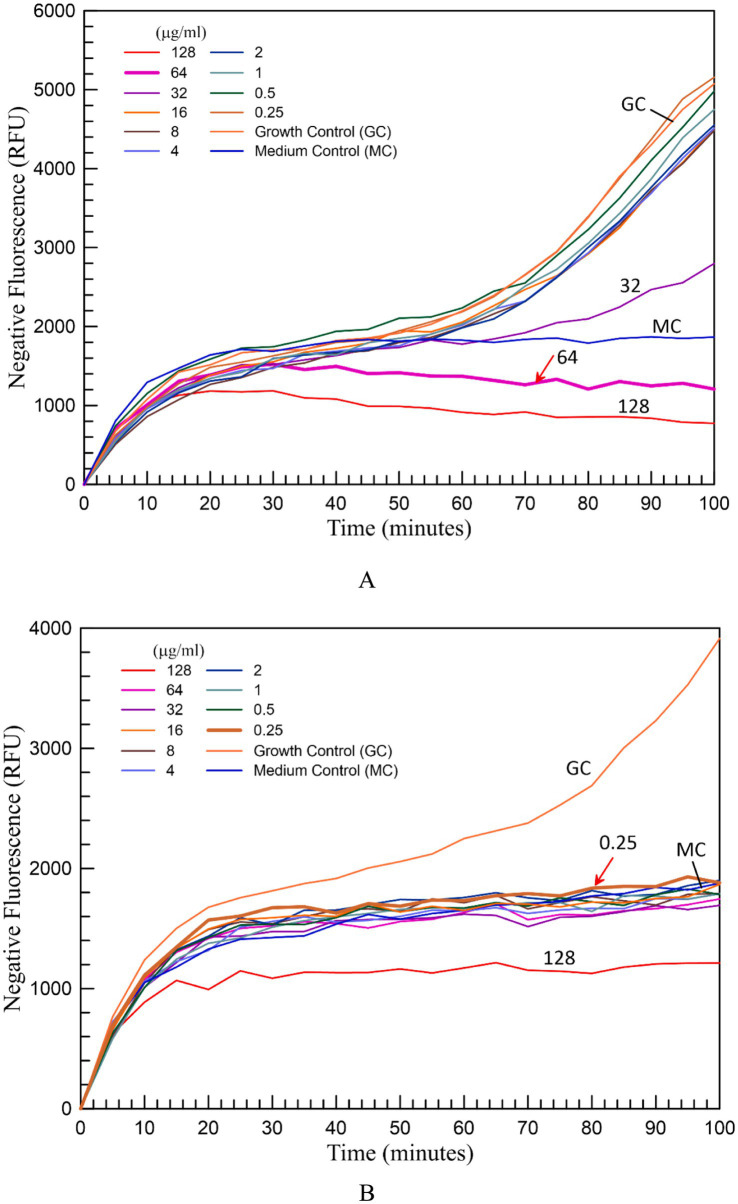
The growth curves shown in figure one are drawn using negative fluorescence values on the “Y” axis, to make easier to determine the MIC values. The growth curves of a colistin-resistant **(A)** and colistin-susceptible **(B)** *K. pneumoniae* strain obtained with the new fluorometric method. The MIC values are indicated by an arrow.

When bacteria started to grow in the growth control well without colistin and in the wells containing different concentrations of colistin, if bacteria were resistant to colistin at these concentrations, it was observed that the fluorescence value started to decrease at around 1 h. In wells where bacteria were inhibited by colistin, there was no change in fluorescence value compared to the uninoculated medium control. In the absolute data, fluorescence values decreased over time (Figure 1). To enhance clarity, the values were inverted and plotted from zero, taking readers’ perception into account. Accordingly, the related graphs were transformed into a growth curve representation, where negative fluorescence values are shown over time (Figure 2). The MIC value of 2 μg/mL or below was considered susceptible to colistin by both standard and fluorometric methods. Graphs showing the growth curves of a colistin-resistant strain with an MIC of 64 μg/mL (Figure 2A) and a colistin-susceptible strain with an MIC of 0.25 μg/mL (Figure 2B) are presented. As can be seen in the graphs, the results can be obtained after 90 min of incubation.

MIC values for colistin of 42 *K. pneumoniae* strains, using standard and fluorometric methods, are shown in Table 1.

**Table 1:** Comparison of MIC values of 28 colistin-resistant and 14 colistin-susceptible *K. pneumoniae* strains with the standard microdilution method and the new fluorometric method.

Sample name	Standard method (μg/ml)	Fluorometric method (μg/ml)
S1	≤0.25	≤0.25
S2	≤0.25	≤0.25
S3	≤0.25	0.5
S4	≤0.25	0.5
S5	≤0.25	1
S6	≤0.25	2
S7	0.5	≤0.25
S8	0.5	0.5
S9	0.5	1
S10	1	≤0.25
S11	1	0.5
S12	1	0.5
S13	2	≤0.25
S14	2	≤0.25
S15	2	0.5
S16	2	1
S17	2	1
S18	2	2
R1	4	4
R2	8	8
R3	8	8
R4	16	8
R5	16	16
R6	32	32
R7	32	32
R8	32	32
R9	32	32
R10	32	64
R11	32	64
R12	64	64
R13	64	64
R14	64	64
R15	64	64
R16	64	128
R17	>128	64
R18	>128	64
R19	>128	>128
R20	>128	>128
R21	>128	>128
R22	>128	>128
R23	>128	>128
R24	>128	>128

Strains S1 to S18 are colistin-susceptible, and strains R1 to R24 are colistin-resistant strains.

In our comparative analysis of minimum inhibitory concentration (MIC) values obtained through the conventional standard method and a novel fluorometric approach, 18 strains were susceptible and 24 were resistant to colistin. All strains identified as resistant or susceptible to colistin by the standard method were also identified as resistant or susceptible to colistin by the fluorometric method correctly. Among the 22 strains studied, an exact match in MIC values was observed; 1-dilution (1/2) difference in 14 strains and 2-dilution (1/4) difference in 6 strains were observed between the two methods. The variation in MIC value was more prominent in susceptible strains with usually low levels of MIC.

### 3.2 Diagnostic performance results

The performance measures used in this study are derived from 2 × 2 contingency table analyses, providing meaningful statistical insight into the model’s ability to correctly classify colistin susceptible and resistant strains, as reported in the literature (Altman and Bland, 1994; Zhou et al., 2011). This methodology is widely accepted and commonly employed in medical literature for evaluating the diagnostic performance of models (Altman and Bland, 1994; Zhou et al., 2011). The Sensitivity, Specificity, PPV, and NPV were all found as 100%. As given in Table 2, the technical agreement of MICs according to within ±1 dilution difference between the two methods was 66.7% for the S profile group (12 of 18 samples), whereas it was 91.7% for the R profile group (22 of 24 samples).

**Table 2:** Technical agreement between standard method and fluorometric method.

Category	Total samples	Agreement	Disagreement	Agreement rate
S	18	12	6	66.7%
R	24	22	2	91.7%
Overall	42	34	8	81.0%

## 4 Discussion

Patients infected with multi-drug resistant *K. pneumoniae* are usually critically ill patients who may have septicemia that requires urgent proper treatment. In these cases, many times colistin is the only choice for treatment. The current colistin reference susceptibility test, which is BMD, requires 24–48 h. Therefore, a rapid colistin susceptibility test is a vital need.

In addition to long incubation time required by the reference colistin susceptibility test, BMD, automated systems commonly used in routine laboratories are not considered valid methods due to their tendency to yield inconsistent results compared to the reference BMD method. Other tests have also been proposed for the determination of colistin susceptibility (such as disk diffusion and gradient strip methods), but due to colistin’s poor diffusion in agar media, these tests exhibit low sensitivity and are not recommended for routine use. Therefore, international committees such as EUCAST recommend the BMD method as the reference method for colistin antimicrobial susceptibility testing (EUCAST (European Committee on Antimicrobial Susceptibility Testing), 2025). In contrast, rapid AST techniques enable patients to be treated more effectively by facilitating timely and proper treatment decisions. This enables a decrease in the side effects of drugs and deaths related to *K. pneumoniae* infections. Additionally, hospital stays are shortened, providing significant gains both in terms of health and economics (Reller et al., 2009).

A study conducted in recent years to detect colistin resistance led to the development of a ready-to-use kit (SensiTest™ Colistin -STC-, Liofilchem, Italy). While this kit facilitates the implementation of liquid microdilution testing in laboratory settings, it still requires a 20-h incubation period (Park et al., 2016). Another test, Colistin-NP (Biomerieux, France), indicates the growth of bacteria in the presence of colistin in 2–4 h using a colorimetric method, yielding qualitative results without determining the MIC value (Nordmann et al., 2016). Additionally, in a study evaluating colistin susceptibility quantitatively, the flow cytometry method was compared with the standard BMD, including 65 susceptible and 109 resistant strains (*E. coli, K. pneumoniae, P. aeruginosa, and Acinetobacter* spp.), which showed sensitivity and specificity of 89 and 94%, respectively. However, the performance of the test for *Acinetobacte*r spp. was found to be low compared to *E. coli*, *K. pneumoniae*, and *P. aeruginosa*. This flow cytometry method provided results within 6 h. Based on these results, the flow cytometry method was recommended for qualitative and quantitative testing of colistin resistance in *E. coli* and *K. pneumoniae* (Ekelund et al., 2021). However, since this method requires high labor, a flow cytometer, and experienced laboratory personnel, it does not offer a cost-effective solution, and it is hard to use in routine laboratory practice. Additionally, another method described in the literature as being low-cost and easy to implement is the polymxin NP method (Malli et al., 2018). However, this method also cannot provide MIC results; it can only report results as colistin-resistant or colistin-susceptible by working with a single concentration. Therefore, there is a need for new methods that can determine MIC, are fast, low-cost, and easy to implement.

In this study, we have developed and tested a novel rapid colistin susceptibility test that gives results within 2 h. This invention not only offers a rapid diagnostic tool for determining the susceptibility of *K. pneumoniae* to colistin but also holds tremendous promise for enabling more timely and effective treatment decisions, especially for critically ill patients. This method, which simplifies the MIC determination process, marks an important step forward in the field of antimicrobial therapy by improving our ability to combat infections caused by *K. pneumoniae* with greater sensitivity and urgency. The reliability of this method should be further evaluated through studies of other bacterial species, such as *E. coli, Pseudomonas aeruginosa*, and *Acinetobacter* spp., which also cause hospital infections in critically ill patients who require treatment with colistin.

In our study the sensitivity, specificity, PPV, and NPV were determined to be 100%. The technical agreement between two methods was 81%. Specifically, the agreement was lower for the susceptible (S) profile group (66.7%), which may be attributable to methodological limitations rather than actual differences in bacterial susceptibility. This issue is particularly pronounced at lower concentrations, since dilution error increases in lower concentrations due to higher number of pipetting to reach these values. These findings suggest that the discrepancies we observed are more likely linked to technical errors inherent to colistin testing, reinforcing current recommendations that broth microdilution should remain the reference method for reliable susceptibility assessment. Furthermore, the fact that high compliance was observed in resistant (R) profile group (91.7%) is a finding confirming this situation due to the higher drug concentration available for bacterial exposure.

In conclusion, the results of this study suggest a promising future for the innovative fluorometric antibiotic susceptibility testing method, particularly in the realm of rapidly determining colistin resistance in *K. pneumoniae*. This advancement has the potential to significantly impact the guidance of treatment strategies for critically ill patients, presenting a valuable tool that aids in ensuring the most effective therapeutic interventions. As we navigate the complex landscape of antimicrobial resistance, the enhanced accuracy and expediency offered by the fluorometric method hold immense promise for improving patient outcomes and advancing the field of infectious disease management.

## Data Availability

The raw data supporting the conclusions of this article will be made available by the authors, without undue reservation.
